# Multiple Administration of Dexamethasone Possesses a Deferred Long-Term Effect to Glycosylated Components of Mouse Brain

**DOI:** 10.3390/neurolint16040058

**Published:** 2024-07-22

**Authors:** Stanislav D. Aladev, Dmitry K. Sokolov, Anastasia V. Strokotova, Galina M. Kazanskaya, Alexander M. Volkov, Svetlana V. Aidagulova, Elvira V. Grigorieva

**Affiliations:** 1 Institute of Molecular Biology and Biophysics FRC FTM, Novosibirsk 630117, Russia; dmit_s95@mail.ru (D.K.S.); anastasia-suhovskih@mail.ru (A.V.S.); kazanskaya10101958@gmail.com (G.M.K.); s.aydagulova@gmail.com (S.V.A.); elv_grig@yahoo.com (E.V.G.); 2 E.N. Meshalkin National Medical Research Center, Novosibirsk 630055, Russia; a_volkov@meshalkin.ru; 3 Laboratory of Cellular Biology, Novosibirsk State Medical University, Novosibirsk 630091, Russia

**Keywords:** glucocorticoid, dexamethasone, glucocorticoid receptor, brain extracellular matrix, glycosylation, proteoglycan, glycosaminoglycan, heparan sulfate biosynthesis

## Abstract

Glucocorticoids are used during glioblastoma treatment to prevent the cerebral edema effect surrounding normal brain tissue. The aim of our study was to investigate the long-term effects of multiple administrations of glucocorticoids onto the glycosylated components (proteoglycans and glycosaminoglycans) of normal brain extracellular matrix and the glucocorticoid receptor (GR, *Nr3c1*) in an experimental model *in vivo*. Two-month-old male C57Bl/6 mice (n = 90) were injected intraperitoneally with various doses of dexamethasone (DXM) (1; 2.5 mg/kg) for 10 days. The mRNA levels of the GR, proteoglycans core proteins, and heparan sulfate metabolism-involved genes were determined at the 15th, 30th, 60th, and 90th days by a real-time RT–PCR. The glycosaminoglycans content was studied using dot blot and staining with Alcian blue. A DXM treatment increased total GAG content (2-fold), whereas the content of highly sulfated glycosaminoglycans decreased (1.5–2-fold). The mRNA level of the heparan sulfate metabolism-involved gene Hs3St2 increased 5-fold, the mRNA level of Hs6St2 increased6–7-fold, and the mRNA level of proteoglycan aggrecan increased 2-fold. A correlation analysis revealed an association between the mRNA level of the GR and the mRNA level of 8 of the 14 proteoglycans-coding and 4 of the 13 heparan sulfate metabolism-involved genes supporting GR involvement in the DXM regulation of the expression of these genes. In summary, multiple DXM administrations led to an increase in the total GAG content and reorganized the brain extracellular matrix in terms of its glycosylation pattern.

## 1. Introduction

Glucocorticoids (GCs) play an important role in normal human physiology and are used in the treatment of numerous pathologies, like inflammatory diseases, neurodegenerative diseases, cancer, COVID-19, etc., either as the main therapeutic agent or as a component of complex therapy [1,2,3]. For glioblastoma, GCs are usually used to prevent the development of cerebral edema, and the regime for taking them may vary significantly in dose and frequency of administration. However, long-term use of GCs in humans results in numerous negative side-effects [4,5,6] such as Cushing’s syndrome [7], neurocognitive impairment, depression, anxiety, mania and delirium [8,9], and neurodegeneration [10].

Various molecular mechanisms involved in the functional effects of GCs in gliomas are described in a recent review by Nafe and Hattingen [11]. These molecular mechanisms include angiogenic, apoptotic, and metabolic pathways such as Wnt and PI3K/AKT/PTEN, as well as regulators of epigenetic processes, including non-coding RNAs and microRNAs. At the same time, GCs’ effects on normal brain tissue remain less studied. It has been shown that dexamethasone (DXM) induces the plasticity of neurons, Schwann cells, oligodendrocytes, astrocytes, and microglia [12] and possesses a neuroprotective effect against brain vestibular damage [13] and neurodegeneration [10].Although, according to the other study, DXM has no effect on normal white matter cells in the contralateral hemisphere of the brain in tumor patients *in vivo* [14]. Most of the described mechanisms are mainly related to the intrinsic properties of normal or glioma cells, whereas GCs’ effects on the brain extracellular matrix (ECM) are still poorly investigated.

The main peculiarity of the brain tissue is that the ECM makes up 20% of total brain mass and mainly consists of hyaluronic acid and complex protein–carbohydrate macromolecules called proteoglycans (PGs) [15,16]. PGs are composed of a core protein and long linear polysaccharide chains of glycosaminoglycans (GAGs) [17,18], which can be sulfated at different positions [19]. The predominant non-sulfated GAG in the brain tissue is hyaluronic acid (HA), while the highly sulfated GAGs include chondroitin sulfate (CS) and heparan sulfate (HS) [19]. While non-sulfated GAGs play a major role in ECM hydration and homeostasis, the sulfated GAGs are responsible for the cell–cell and cell–matrix interactions, the structural organization of the matrix, the main signaling cascades, and cell functions such as adhesion, migration, and proliferation [20,21].

There is very little information about the effect of GCs on the PGs and GAGs in normal brain tissue [22]. In particular, single injections of methylprednisolone led to a decrease inthe mRNA levels of neurocan and phosphacan in cultured astrocytes both in vitro and in a model of acute spinal cord injury *in vivo* [23]. A local delivery of DXM-contained silicone probes into the brains of Sprague–Dawley rats decreases the CS content in the rat brain tissue after probe implantation [24]. The chronic stress and multiple DXM injections reduce the mRNA level of the core basement membrane proteoglycan agrin and impair the AQP4-mediated glymphatic transport in the brain via GR signaling [25]. Taken together, according to the literature data, DXM reduces the mRNA level of PGs in brain cell cultures in vitro and the spinal cord and brain tissue *in vivo*.

Changes in the mRNA level of PG after a single injection of DXM depending on the dose of the drug were also studied. A single administration of DXM to Wistar rats at a dosage of 2.5 or 5 mg/kg increases the mRNA level of some PG core proteins in the rat’s normal brain tissue *in vivo* in a dose-dependent manner. In our previous work, it was shown that lower DXM doses (0.1, 1 mg/kg) affect the content of protein molecules of these PGs; however, a higher dose (5 mg/kg) affects the level of transcriptional activity of the corresponding genes. Along with the elevation inthe mRNA levels of PG core proteins, DXM decreases the content of carbohydrate CS and HS chains, suggesting deterioration of the structure of complex PG macromolecules in brain ECM [26].

The changes in PG/GAG composition after DXM treatment have significant functional effects on experimental models of glioblastoma relapse. It was shown that DXM-induced changes in the composition and content of brain PGs result in accelerated adhesion, proliferation, and invasion of U87 glioblastoma cells into brain organotypic slices ex vivo and more active growth of U87 xenografts in SCID mouse brain *in vivo*. These facts demonstrate that chemotherapy-induced changes in the brain ECM can impact glioblastoma relapse development [27].

Taken together, these data demonstrate that even a single administration of GCs is able to deteriorate a composition and/or structure of brain ECM, but this effect is very fast and returns to the control values in a few days after GC injection. However, the prolonged effects of the chronic administration of GCs towards the normal brain ECM are still unknown.

The aim of this work was to study the effects of long-term (15–90 days) multiple administrations of DXM on the mRNA levels of PG core proteins, HS-metabolism-involved genes, and GAGs content in the normal brain tissue. As the functional effects of DXM areimplemented through its interaction with the glucocorticoid receptor (GR) encoded by the *Nr3c1* gene (Nuclear Receptor family 3, member 1), we aimed to estimate the mRNA level of *Nr3c1* in mouse brains as well and investigate its association with the mRNA levels of the PG-metabolism-involved genes.

## 2. Materials and Methods

### 2.1. Animals

All studies were performed on male C57Bl/6 mice, 7–8 weeks old, weighing 22–30 g. The animals (n = 90) were obtained from the Institute of Cytology and Genetics (Novosibirsk, Russia) and were kept in polycarbonate cages with free access to food and water. The cells were placed in a ventilated room with a 12/12 h light/dark cycle, 25 ± 1 °C temperature, and 50–60% humidity.The animals were randomly assigned to groups;simple randomization using random number generation was used.The control group (n = 10) consisted of animals that received a single injection of saline. The rest of the mice (n = 80) received ten intraperitoneal injections of DXM (KRKA Pharma, Slovenia) at a dosage of 1 and 2.5 mg/kg (20 animals in each group). The terms for removing animals from the experiment for each of the DXM dosages were 15, 30, 60, and 90 days. The animals were sacrificed by decapitation using a guillotine according to the animal euthanasia guidelines (National Research Council Committee for the Update of the Guide for the Care and Use of Laboratory, 2011).The brain from each animal was collected;one hemisphere was placed in an RNALater solution (Invitrogen; ThermoFisher Scientific, Inc., Waltham, MA, USA) for reverse transcription–quantitative PCR (RT–qPCR), Westernblot, and dot-blot analysis.The other hemisphere was incubated in a 10% neutral buffered formalin for 48 h at room temperature and used to prepare paraffin blocks.All procedures with experimental animals were carried out in accordance with the Directive of the Council of the European Community 2010/63/EU and approved by the local committee on biomedical ethics of the Institute of Molecular Biology and Biophysics FRC FTM(approval no. N4/2017 from 23.06.2017; Novosibirsk, Russia).

### 2.2. Real-Time RT–PCR

Total RNA was isolated using a QIAzol Lysis Reagent (Qiagen, Hilden, Germany). The reverse transcription was performed using a RevertAid H Minus First Strand cDNA Synthesis Kit (Thermo Fisher Scientific, Agawam, MA, USA). The real-time PCR for GR-, PG-, and HS-biosynthetic enzymes was performed on a CFX90 instrument (BioRad, Hercules, CA, USA) using a HS-qPCR SYBR Blue (Biolabmix, Moscow, Russia) and primers (Supplemental Table S1). The expression level was determined by the formula, (2^ΔCt^) × 1000, where ΔCt = Ct (Gapdh)−Ct (gene) for each sample (animal).

### 2.3. Western-Blot

The mouse brain tissue was homogenized in a RIPA buffer (Thermo Fisher Scientific, Waltham, MA, USA) with protease inhibitors complete Mini (Roche, Mannheim, Germany). Vertical electrophoresis was performed in a 10% polyacrylamide gel; proteins were transferred to an Immobilon-PVDF membrane (Millipore, Darmstadt, Germany) and blocked using 5% milk powder (BioRad, Hercules, CA, USA) in a PBST buffer for 60 min. The membranes were then incubated with primary rabbit antibodies of GR proteins (ab183127, Abcam, Cambridge, UK, 1:5000) or Gapdh (ab181602, Abcam, Cambridge, UK, 1:15,000) and then with secondary antibodies (ab3578, Abcam, Cambridge, UK, 1:10,000) for 60 min at room temperature. The signal was visualized using Amersham ECL Prime (Cytiva, Marlborough, MA, USA), detection was performed in Bio Rad Chemidoc (BioRad, Hercules, CA, USA), and the obtained data were processed using ImageLab 6.0.1 (BioRad, Hercules, CA, USA).

### 2.4. Alcian Blue Staining for a Total and Highly Sulfated GAG Content

GAGs content in the samples was determined by staining paraffin sections with Alcian blue with different pH (pH = 2.5 for total GAGs and pH = 1.0 for highly sulfated GAGs) (BioVitrum, Novosibirsk, Russia), according to the protocol specified in the manufacturer’s instructions, with the addition of Ehrlich’s hematoxylin. The stained preparations were analyzed using an AxioScope A1 microscope (Zeiss, Oberkochen, Germany). The resulting images were processed for quantitative analysis using the chromoanalytical algorithms of the ZEN Pro (Carl Zeiss, Oberkochen, Germany) to determine the ratio of the tissue area that gives a specific staining signal to the total image area, expressed as a percentage.

### 2.5. Dot-Blot Analysis for HS and CS Content

The mouse brain tissue was homogenized in the RIPA buffer (ThermoFisher Scientific, Agawam, MA, USA) with protease inhibitors complete Mini (Roche, Mannheim, Germany), and the resulting lysates were applied to a PVDF membrane activated in 95% ethanol (Millipore Immobilon-P, Darmstadt, Germany), blocked in 5% fat-free milk in the PBST (BioRad, Hercules, CA, USA), incubated with primary antibodies of HS (MAB2040, Millipore, Darmstadt, Germany, 1:100) or CS (8035, Sigma-Aldrich, St. Louis, MO, USA, 1:100) or GR (ab183127, Cambridge, Abcam, UK), and then with secondary antibodies (150077, Thermo Fisher Scientific, Agawam, MA, USA, 1:1000) for 60 min at room temperature. The signal was developed using Amersham ECL Prime (Cytiva, Marlborough, MA, USA), detection was performed in BioRadChemidoc, and the data obtained were processed using ImageLab 6.0.1 (BioRad, Hercules, CA, USA).

### 2.6. Statistical Analysis

The statistical analysis of the obtained data was carried out using MS Excel 7.0 (Microsoft, Redmond, WA, USA) and Origin Pro 8.5 software (OriginLab, Northampton, MA, USA). Statistical significance was determined using one-way analysis of variance (ANOVA) with Fisher’s least significant difference posthoc test. The correlation analysis was performed using Spearman test. Multiple comparison adjustments were applied with Benjamini–Hochberg correction. The normal distribution was checked in the Origin 8.5 (OriginLab, Northampton, MA, USA) using the Kolmogorov–Smirnov test. *p* < 0.05 was considered to indicate a statistically significant difference.

## 3. Results

### 3.1. DXM Affects Content of Total and Sulfated GAGs in the Mouse Brain Tissue

**Figure 1:**
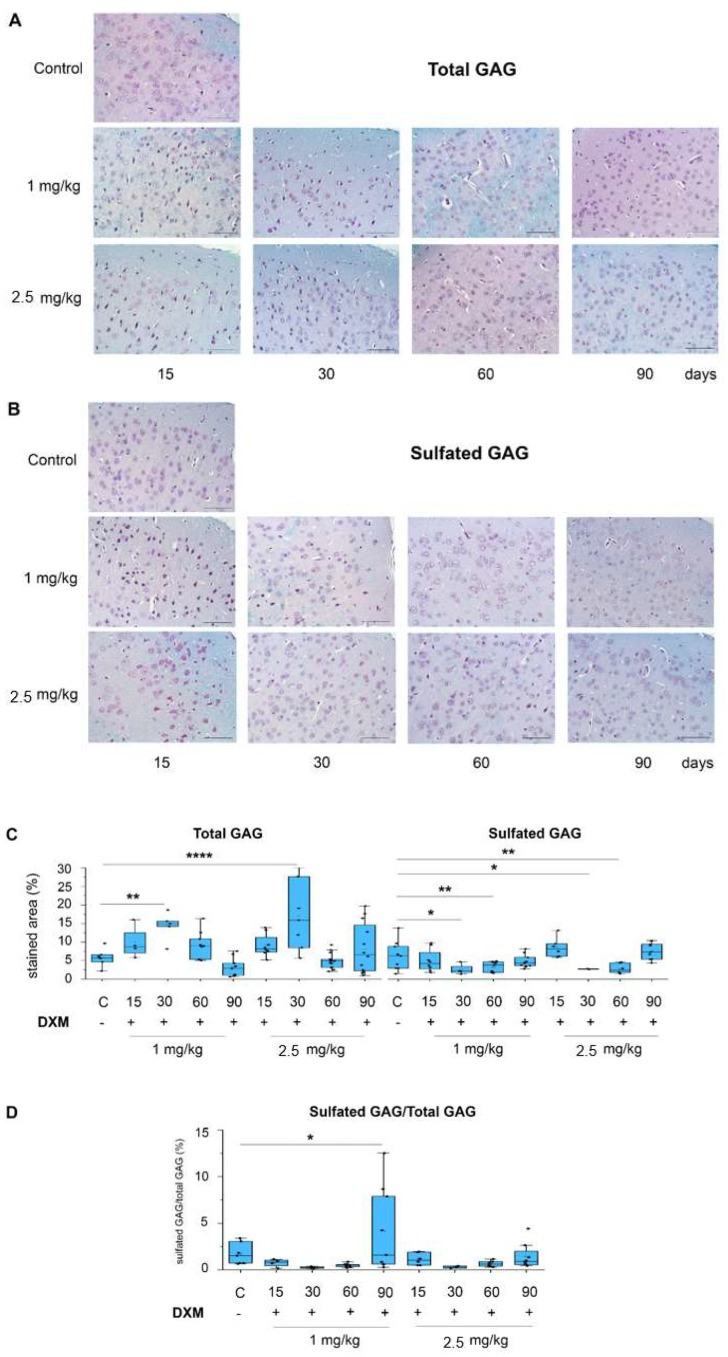
The content of GAGs in the cerebral cortex of mice upon multiple DXM administration.DXM doses of 1 and 2.5 mg/kg were used, the studied parameters were determined at 15, 30, 60 and 90 days after last DXM injection by Alcian blue–Ehrlich’s hematoxylin staining. (**A**) Microphotographs of the total GAG staining in the mouse cerebral cortex (pH = 2.5). (**B**) Microphotographs of the sulfated GAG staining in the mouse cerebral cortex (pH = 1.0). (**C**) Quantitative analysis of the total and sulfated GAGs content in mouse brain tissue before and after DXM administration. The content is represented by the percentage of the stained area in the sample. Control mice treated with saline. (**D**) Sulfated GAGs/total GAGs proportion. “C”—“control group”; “15”, “30”, “60”, “90”days after DXM administration. Medians and IQRs are presented. ANOVA + Fisher’s least significant difference test; *—*p* < 0.05, **—*p* < 0.01, ****—*p* < 0.0001. Magnification ×400. Scale bar 50 μm. DXM: dexamethasone.

Since GAG carbohydrate chains contribute significantly to the functional activity of complex PG macromolecules, the DXM effects on the GAG content in the mouse brain tissue were studied by staining the paraffin sections of the brain tissue with Alcian blue with pH = 2.5 (total GAGs) and pH = 1 (highly sulfated GAGs) (Figure 1).

Multiple administrations of DXM for 10 days resulted in a significant increase in the total GAG content reaching the maximal levels at day 30 (+ 2 fold, t(65) = 3.09, *p* = 0.003 for 1 mg/kg DXM; t(65) = 4.4, *p* = 3.82 × 10^−5^ for 2.5 mg/kg DXM). At the same time, the content of highly sulfated GAGs demonstrated an opposite trend, being decreased (−1.5–2 fold, t(57) = 2.56, *p* = 0.01 at 30 days for 1 mg/kg DXM; t(57) = 2.8, *p* = 0.007 at 60 days for 1 mg/kg DXM; t(57) = 2.21, *p* = 0.03 at 30 days for 2.5 mg/kg DXM; t(57) = 2.73, *p* = 0.008 at 60 days for 2.5 mg/kg DXM), with maximum levels at days 30–60 (Figure 1A–C). This suggests that the observed increase in the total content of GAGs occurred mainly due to significant increase in the content of non-sulfated GAGs (primarily hyaluronic acid), whereas the content of highlysulfated GAGs (HS, CS) decreased. Although all the studied parameters returned to the control levels by day 90, there was a prolonged-enough (1–1.5 months) time period of deteriorated brain ECM composition which could potentially be meaningful for the brain’s functionality. The proportion of sulfated GAGs/total GAGs was statistically increased at day 90 after multiple administrations of DXM (t(57) = 2.43, *p* = 0.019) (Figure 1D).

**Figure 2:**
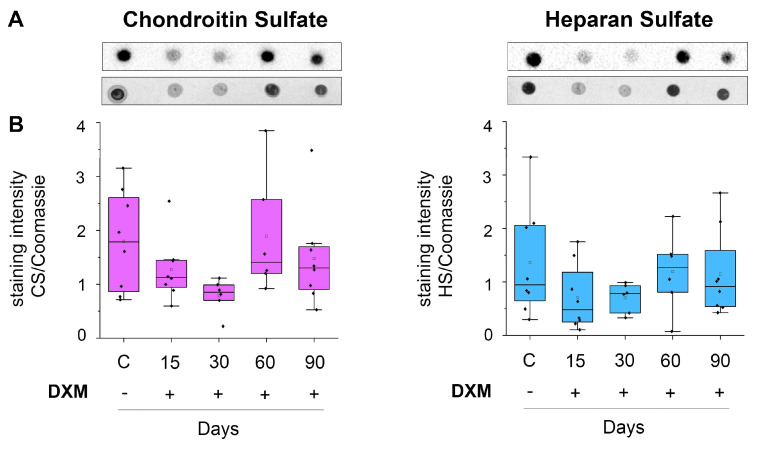
The content of the carbohydrate molecules of CS and HS in the cerebral cortex of mice upon multiple DXM administration. (**A**) Original microphotographs of dot blot staining for CS and HS polysaccharide with anti−HS and anti−CS antibodies. (**B**) Quantitative analysis of CS and HS content. Medians and IQRs are presented. ANOVA + Fisher’s least significant difference test; DXM: dexamethasone.

Since highly sulfated GAGs are represented by HS and CS, a dot-blot analysis was performed using specific antibodies of CS and HS to assess the effect of the repeated administration of DXM onto the CS and HS content in the mouse brain tissue (Figure 2).

DXM administration has a tendency to decrease the content of CS at day 15–30 and HS at day 15, although this effect was not statistically significant (for CS F(4, 35) = 1.85, *p* = 0.14; for HS (F(8, 35) = 1.15, *p* = 0.35). This effect was quite long-lasting, at least up to 90 days after the last DXM administration (Figure 1B) and coincided with the observed earlier decrease in highlysulfated GAGs at the 30th day after multiple DXM injections (Figure 1C).

Due to the fact that the content of carbohydrate macromolecules of HS completely depends on the functioning of the system of its biosynthetic enzymes, we have profiled the expression of the corresponding genes.

### 3.2. DXM Down-Regulates Transcriptional Activity of HS-Biosynthesis-Involved Genes

**Figure 3:**
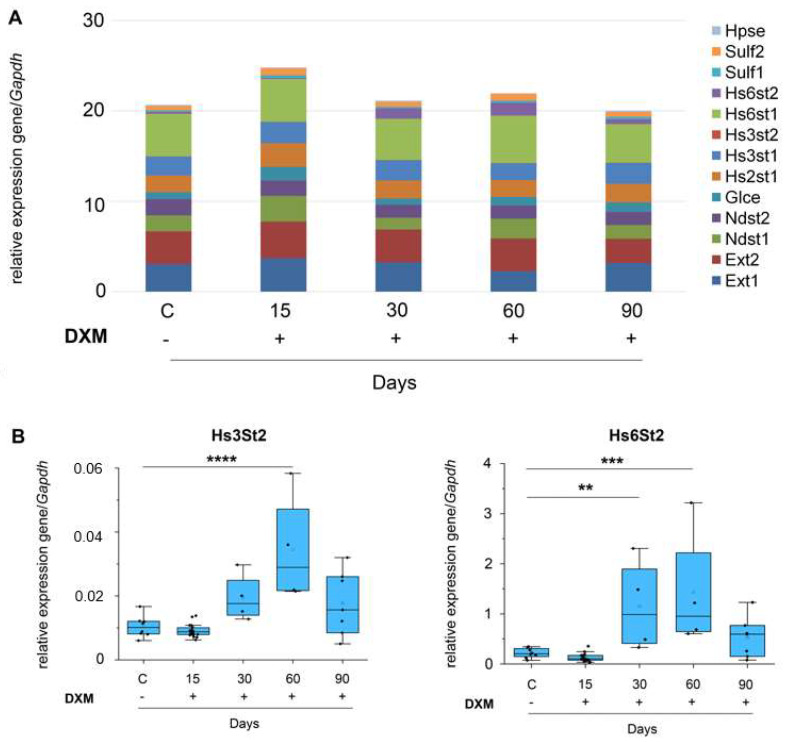
DXM effects on the mRNA level of HS−biosynthesis-involved genes in mouse brain tissue. DXM doses of 1 and 2.5 mg/kg were used, and the studied parameters were determined 15, 30, 60 and 90 days after last DXM injection. (**A**) Overall transcriptional activity of HS−biosynthetic system. Stacked columns compare the contribution of each value to the total across categories. (**B**) The mRNA level of the most affected individual genes. RT–PCR analysis, intensity of the amplified DNA fragments for each gene normalized to that of Gapdh. Control: mice treated with saline solution. Medians and IQRs are presented. ANOVA + Fisher’s least significant difference test; **—*p* < 0.01, ***—*p* < 0.001, ****—*p* < 0.0001. DXM: dexamethasone.

The effect of DXM on the mRNA level of HS-biosynthesis-involved genes was studied using the real-time RT–PCR (Figure 3, Supplemental Table S2).

Since we have two doses of DXM, 1 mg/kg and 2.5 mg/kg, that are close one to another in terms of pharmacokinetics, we analyzed the obtained results by combining the relevant 1 mg/kg and 2.5 mg/kg groups. The overall transcriptional activity of the HS biosynthetic system demonstrated a tendency for dose-dependent activation 15 days after DXM injections, which was replaced by its temporal suppression at 30–60 days of the experiment (Figure 3A). This down-regulation was due to the decrease in the HS content in this time interval (Figure 2B). Although, most of the demonstrated changes were not statistically significant because of the large standard deviation of the parameters in the individual animals (discussed in the Discussion section). However, several differences have been identified for some genes. The Hs3St2 mRNA level was increased on day 60 (t(41) = 5.6; *p* = 0.0006) and the Hs6st2 mRNA level was increased on day 30 (t(41) = 3.24; *p* = 0.0003) and day 60 (t(41) = 4.21; *p* = 0.01) after DXM treatment.

### 3.3. DXM Administration Does Not Affect the Expression of PG Core Protein-Coding Genes

**Figure 4:**
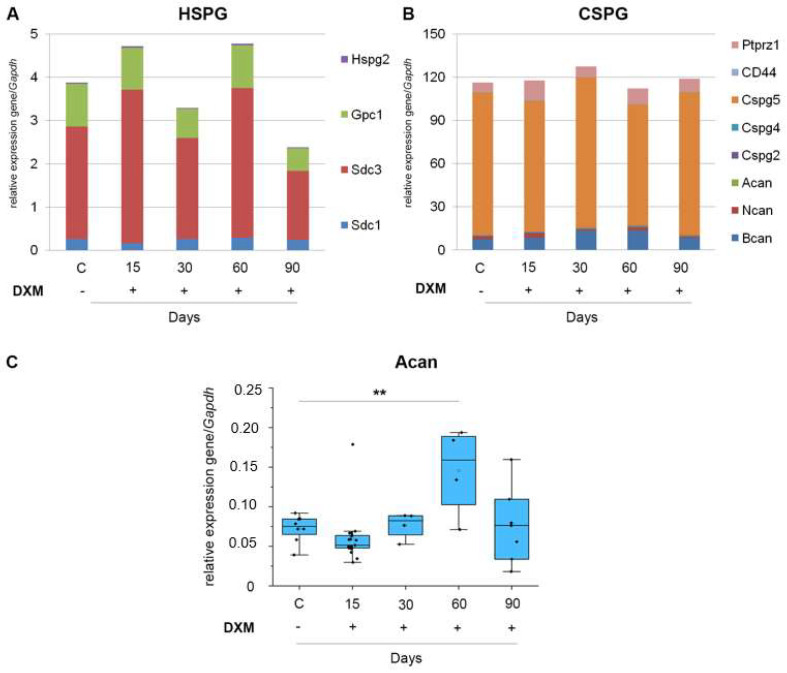
The DXM effect on the mRNA level of HSPG and CSPG core proteins in mouse brain tissue. DXM doses of 1 and 2.5 mg/kg were used; the studied parameters were determined at 15, 30, 60 and 90 days after last DXM injection. The overall transcriptional activity of HSPG (**A**) and CSPG-coding genes (**B**). Stacked columns compare the contribution of each individual PG to a total across categories. RT–PCR analysis, intensity of the amplified DNA fragments for each gene normalized to that of Gapdh. (**C**) The mRNA level of the most affected individual gene. RT–PCR analysis, intensity of the amplified DNA fragments for each gene normalized to that of Gapdh. Control: mice treated with saline solution. Medians and IQRs are presented. ANOVA + Fisher’s least significant difference test; **—*p* < 0.01. DXM: dexamethasone.

To investigate whether the demonstrated tendency towardsthe down-regulation of CS/HS content upon DXM pressure depends also from the mRNA level of CSPG and HSPG core proteins, their transcriptional profiling was performed (Figure 4, Supplemental Table S3).

The mRNA level of the studied HSPG and CSPG core proteins responded to the multiple DXM administration, demonstrating changes at the different DXM doses and time-points (near 50% decrease in HSPG’s mRNA level at 30 and 90 days for 1 mg/kg DXM and 25% decrease in CSPG’s mRNA level at day 60 for 2.5 mg/kg DXM). The HSPG’s mRNA level was not changed after multiple DXM injections. Among CSPGs, the mRNA level of aggrecan increased after multiple administrations of DXM by day 60 (t(41) = 3.5; *p* = 0.0049).

At the same time, a tendency towards an increase in the HSPG and CSPG overall mRNA levels by day 90 (only for DXM dose 2.5 mg/kg) was observed but, since this was already quite a long time after the DXM administration, this effect may be associated with other physiological or age-related changes in the mouse brain.

However, we paid a special attention to the very high standard deviations of the studied parameters in each experimental group. Possibly, that reflects a deterioration of the regulation of these mRNA levels under the DXM pressure. Bearing in mind that GCs’ effects depend on the mRNA level/content of GR, and this transcription factor has responsive elements in the promoter regions of the most studied genes, we investigated the mRNA level of GR in the DXM-affected mouse brain tissue and its correlation with the mRNA levels of PGs core proteins and HS biosynthetic enzymes.

### 3.4. DXM Decreases GR mRNA Level of Expression in Mouse Brain Tissue

**Figure 5:**
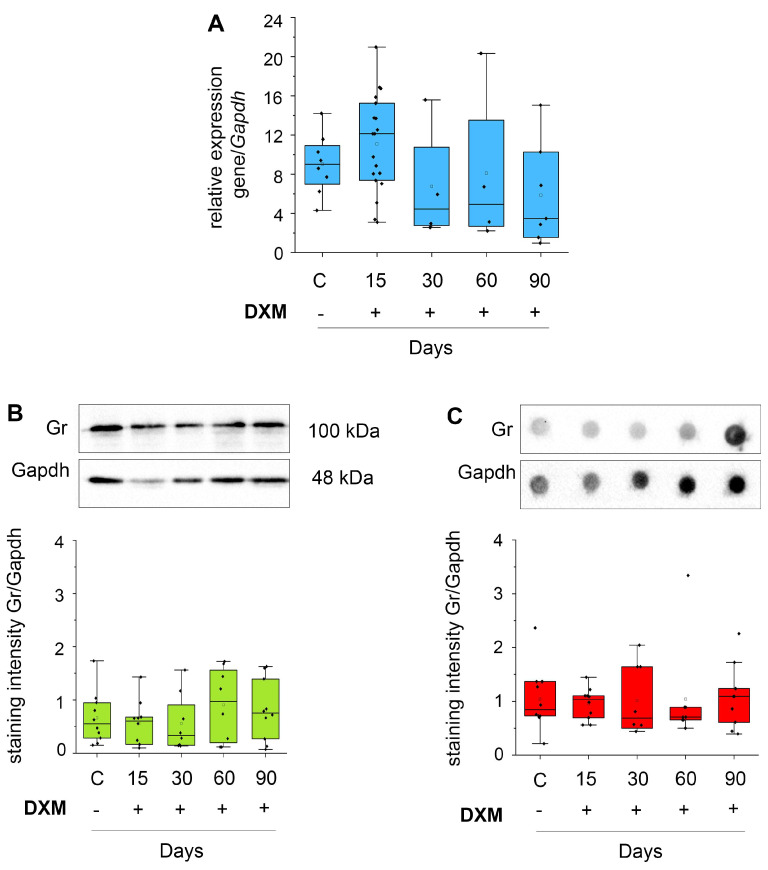
DXM effectson the mRNA level of GR in mouse brain tissue. DXM doses of 1 and 2.5 mg/kg were use; the studied parameters were determined 15, 30, 60, and 90 days after the last DXM injection. (**A**) The mRNA level of GR. RT–PCR analysis, intensity of the amplified DNA fragments for each gene normalized to that of Gapdh. (**B**,**C**) GR protein content according Western−blot (**B**) and dot blot (**C**) analyses. Original microphotographs (upper parts) and quantitative analysis (lower parts). Medians and IQRs are presented. ANOVA + Fisher’s least significant difference test. DXM: dexamethasone.

The mRNA level of GR and the content of GR protein were determined in the mouse brain tissue using the real-time RT–PCR, dot blot, and Western-blot analyses (Figure 5).

It was shown that the mRNA level of GR didnot change (F(4,41) = 1.64, *p* = 0.19) after DXM administration (Figure 5A). The GR protein content was more stable and did not demonstrate any changes upon DXM administration (for Western blot F(4,45) = 0.72, *p* = 0.58; for dot-blotF(4,45) = 0.07, *p* = 0.99) (Figure 5B,C). However, the standard deviation was also very high in all experimental groups.

**Figure 6:**
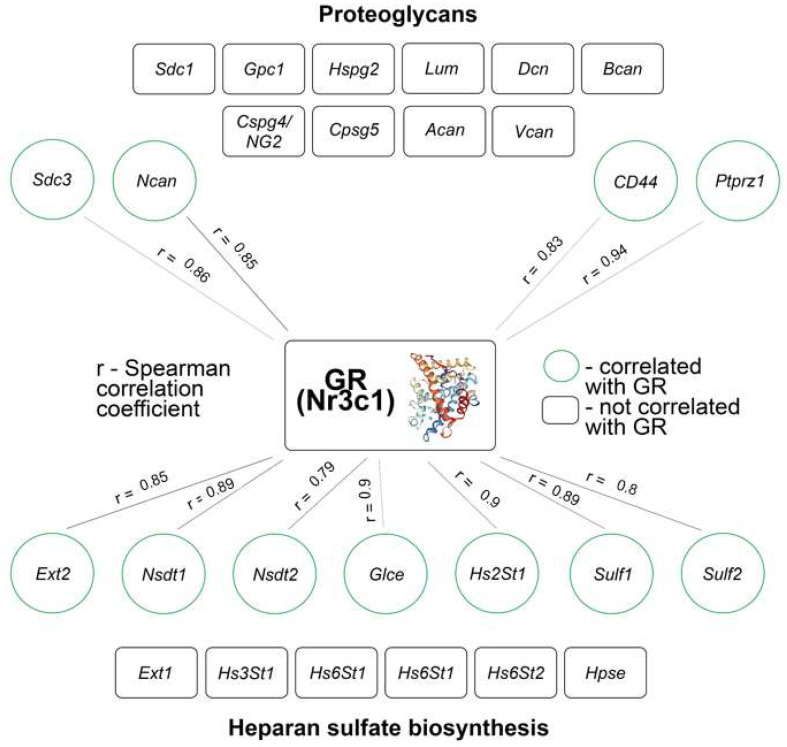
Correlation analysis of mRNA level of GR with PG core proteins and HS-biosynthesis-involved genes in normal mouse brain tissue. The numerical indicator is Spearman correlation coefficient; values with statistically significant correlation are highlighted in green color (*p* < 0.05).

To check the hypothesis on the possible correlation between the mRNA level of GR and the mRNA level of PG core proteins and HS-metabolism-involved genes, the Spearman correlation coefficient was determined for normal mouse brain tissue (Figure 6).

The correlation analysis showed that normal brain tissue is characterized by a strong correlation between mRNA level of GR and that of PG’s genes (4 out of 14 genes had a strong Spearman correlation, r = 0.79–0.94, *p* < 0.05) and some HS-metabolism-involved genes (7 out of 13 genes had a strong Spearman correlation, r = 0.79–0.91, *p* < 0.05). These data are one of the arguments about the involvement of GR in the transcriptional regulation of the genes involved in PGs’ biosynthesis.

## 4. Discussion

The aim of this work is to study the effects of long-term multiple administrations of DXM on the mRNA levels of PG core proteins, HS-metabolism-involved genes, and GAGs content in normal brain tissue. The study of carbohydrate chains is a complex task and requires the use of time-consuming methods, for example, mass-spectrometry. An alternative is to use semi-quantitative methods using specific dyes, such as Alcian blue [28]. Alcian blue, depending on the pH of the dye solution, reveals highly sulfated (pH = 1) or total (pH = 2.5) GAGs. Another possible approach to studying GAGs is the use of specific antibodies of the carbohydrate molecules heparan sulfate [29] and chondroitin sulfate [30]. Since GAG carbohydrate chains are highly charged heterogeneous molecules, the dot-blot analysis was chosen as an alternative to polyacrylamide gel electrophoresis and Western-blot for the study.

The demonstrated decrease in highly sulfated GAGs content in the mouse brain tissue reaching a minimum at 30–60 days after multiple DXM administration indicates that ECM reactivity may be weakened and intercellular contacts and signaling pathways are hindered for quite a long time.

This result extends our previous data on the single DXM injection (monitored for 1–10 days after the injection) for more distant time periods. Although an increase in the sulfated GAGs content in the C57Bl/6 mice brain after the single DXM administration was observed [27], multiple DXM doses for a long period led to the suppression of sulfated GAGs deposition. Interestingly, a similar effect was observed upon incubation of DXM with organotypic culture of rat brain *ex vivo* and after multiple (1 week, daily) DXM administrations to Wistar rats, where DXM reduced the level of CS and HS molecules (2–2.5-fold) in both the cerebral cortex and hippocampus [26]. Multiple administration of 1 mg/kg DXM in the glioblastoma relapse experimental SCID mice model *in vivo* (3 cycles × 5 injections) also resulted in a decrease in CS content and HS in both normal and paratumorous brain tissues [31,32]. A decrease in the CS content was observed in the brains of Sprague–Dawley rats upon local implantation of DXM inside the silicone probes for prolonged control release at as early as 1 week of probe implantation [24]. Taken together, these results demonstrate an inhibitory activity of DXM towards the GAGs content in normal brain tissue for different rat (Wistar, Sprague–Dawley) and mice (C57Bl/6, SCID) strains.

To study the mRNA level of PGs after long-term DXM treatment, *Gapdh* was selected as a housekeeping gene. Previously in the literature, it was shown that this gene has stable expression after long-term glucocorticoid treatment. According to the available data, *Gapdh* could be used as a reference gene to normalize target gene expression in the cortex and hippocampus in rats treated with DXM [33] and in the fetal rat hippocampus [34].The observed decrease in the GAGs content may be due to down-regulation of the mRNA level of PG core protein-coding genes. In this study, we observe statistically significant changes in the mRNA level of aggrecan by 60 days after DXM treatment. However, there are published data on the ability of GCs to affect the mRNA level of some PG core proteins. DXM increased the mRNA levels of biglycan (−2.7-fold), glypican-1 (−3.5-fold), syndecan-1 (−4.3-fold), and versican (−3.1-fold) in the normal brain tissue of SCID mice [27]. High-dose DXM treatment (2.5 mg/kg) resulted in an increase inthe mRNA levels of syndecan-1 (−4-fold), glypican-1 (−3-fold), brevican (−7-fold), and decorin (−2-fold) in the normal brain tissue of Wistar rats [26]. On the other hand, there are a few works demonstrating opposite data. Single treatment with methylprednisolone (at a dosage of 10 and 50 μM) decreases mRNA levels of CSPG neurocan (−1.5-fold) and phosphacan (−2-fold) in AMPA + cyclothiazide-induced primary cultured astrocytes from Sprague–Dawley rats. In the rat acute spinal cord injury *in vivo* model, a single injection of methylprednisolone at a dosage of 30 mg/kg decreases the neurocan mRNA level (−2–3-fold) in the rats on the 1st day after treatment, demonstrating that methylprednisolone is able to suppress the mRNA level of some CSPG core proteins [23]. In an experimental model of the chronic unpredictable mild stressmice model of depression, multiple DXM injections (at a dosage of 5 mg/kg) to C57Bl/6 mice for 6 consecutive weeks reduced the mRNA level of the core basement membrane protein agrin by 1.5 times [25]. Based on these data, we can suggest that the effect of DXM on the mRNA level of PGs is ambiguous, and the DXM-induced decrease inthe CS and HS content in brain tissue may not change due to a decrease in the mRNA level of some PG core proteins.

We profiled the mRNA level of the main genes involved in HS biosynthesis and post-synthetic modification. The lack of significance for the most genes was mainly because of a high standard deviation and seems due to high heterogeneity of the studied parameters in the individual animals. It has been shown that Hs3St2 demonstrated increase on day 60, and Hs6St2 30 and 60 days after DXM administration. There are published data which show that a single administration of DXM can induce an increase in some HS biosynthetic enzymes (Ndst1, Glce, Hs2St1, Hs6St1, Sulf1, Sulf2), but this effect was short-term (3 days after DXM treatment injection) [31]. However, another study described that the transcriptional activity of HS biosynthetic systems was not affected by DXM administration in the normal brain tissue of SCID mice [32]. Taken together, the mRNA level of the HS biosynthesis system practically does not respond to the DXM treatment.

We also observe that the mRNA level of GR (Nr3c1) has no significant changes along with high SD. However, a pairwise correlation analysis revealed high correlation of the mRNA level of GR with 4 out of 14 PG core protein-coding genes (Spearman coefficients r = 0.79–0.94) and 7 out of 13 HS-metabolism-involved genes (Spearman coefficients r = 0.79–0.91) in the normal mouse brain tissue. This may be explained by the fact that the promoters of these genes contain GR-binding sites, or they are regulated by GR indirectly through other factors [35]. These results stay in line with our previously published work on the short-term effects of the single DXM injection to C57Bl/6 mice, where a high correlation between GR- and PG-metabolism-involved genes (20 out of 28 genes had a Spearman coefficient with the mRNA level of GR r = 0.81–0.97) was shown for the normal brain tissue. DXM administration led to the disappearance of this correlation in a dose-dependent manner, with almost no correlation at the highest DXM dose (by day 10, 0–2 genes out of 28 had a correlation coefficient of r = 0.82–0.87) [27]. Interestingly, the mRNA level of GR did not correlate with the mRNA levels of HS-metabolism-involved genes in the experimental U87 tumors grown in SCID mice (1 gene out of 13, r = 0.98), but DXM administration contributed to the appearance of correlation (4 gene out of 13, r = 0.93–0.96) [32]. In any case, the observed correlation was only positive for all described experiments.

From this point of view, one could suppose that the observed DXM-induced decrease in GAG content and transcriptional activity for at least some HS-biosynthesis-involved genes should be accompanied by the decrease in the mRNA level of GR. In this study, we did not detect significant changes in the mRNA level of GR, but indirect support is present in the other studies. Chronic DXM administration at a dosage of 5 mg/kg for 7–28 days resulted in a 2-fold decrease in the level of GR content (*p* < 0.01) in the mouse brain frontal cortex and hippocampus, and by 28 day, the content of GR was the lowest [30]. Chronic DXM exposure (5 μΜ for 3 days) significantly reduced the mRNA level of GR in the cultured Sprague–Dawley rat’s hippocampal neurons [31]. These data can indirectly support GR involvement in the transcriptional regulation of PG-metabolic genes.

## 5. Conclusions

GCs are actively used for the treatment of numerous diseases as effective anti-inflammatory drugs. The most investigated molecular mechanism of their negative side-effects is related to the trans-activation of the expression of multiple genes, among which, apparently, there are also genes involved in the biosynthesis of PG core proteins and HS-biosynthetic enzymes. The presented results on the ability of chronic DXM administration to simultaneously increase the content of non-sulfated GAGs (HA) and decrease that of highly sulfated GAGs (HS, CS) indicate that the structure of the brain ECM will be significantly changed for a long time period (up to 45 day in the mouse experimental model). These changes may represent an additional molecular mechanism of side effects of GCs, which will allow us to start searching for potential protective drugs to prevent the deterioration of brain ECM structure during GC therapy and weaken at least some of the known side effects of DXM on normal brain tissue.

## Data Availability

The original contributions presented in the study are included in the article. Further inquiries can be directed to the corresponding author.

## Supplementary Materials

The following supporting information can be downloaded at: https://www.mdpi.com/article/10.3390/neurolint16040058/s1, Table S1: Primer sequences for mouse PG core proteins- and HS biosynthetic enzymes-coding genes; Table S2: Expression of the HS-metabolism-involved genes in the mouse cerebral cortex upon multiple DXM administration; Table S3: Expression of PG core proteins-coding genes in the mouse cerebral cortex upon multiple DXM administration.
