# Supplementary material for: Multiple Administration of Dexamethasone Possesses a Deferred Long-Term Effect to Glycosylated Components of Mouse Brain
Source: Neurol Int. 2024 Jul 22;16(4):790–803. doi: 10.3390/neurolint16040058 (PMC11270268; doi:10.3390/neurolint16040058)
Supplement: Supplementary file 1 [file neurolint-16-00058-s001.zip › Supplementary Table 1 R5.pdf]

Supplementary Table 1. Primer sequences for mouse PG core proteins-and HS biosynthetic enzymes-coding genes.

Name of the gene	Gene	Primer sequences
Syndecan-1	Sdc1	F 5′-GGTCTGGGCAGCATGAGAC-3′<br>R 5′-GGAGGAACATTTACAGCCACA-3′
Syndecan-3	Sdc3	F 5′-GGTCAGGCTCTGGCTACTTC-3′<br>R 5′-GGGACTGGTGACTGGTTCAG-3′
Glypican-1	Gpc1	F 5′-CTTTAGCCTGAGCGATGTGC-3′<br>R 5′-GGCCAAATTCTCCTCCATCT-3′
Perlecan	Hspg2	F 5′-CCGTGCTATGGACTTCAACG-3′<br>R 5′-TGAGCTGTGGAGGGTGTATG-3′
Decorin	Dcn	F 5′-CCCCTGATATCTATGTGCCC-3′<br>R 5′-GTTGTGTCGGGTGGAAAATC-3′
Biglycan	Bgn	F 5′-GCCTGACAACCTAGTCCACC-3′<br>R 5′-CAGCAAGGTGAGTAGCCACA-3′
Lumican	Lum	F 5′-GCTCGAGCTTGATCTCTCCT-3′<br>R 5′-CAGTGGTCCCAGGATCTTACA-3′
Brevican	Bcan	F 5′-GTGGAGTGGCTGTGGCTC-3′<br>R 5′-AACATAGGCAGCGGAAACC-3′
Neurocan	Ncan	F 5′-CCAGCGACATGGGAGTAGAT-3′<br>R 5′-GGGACACTGGGTGAGATCAA-3′
Aggrecan	Acan	F 5′-TTCGCAGGGATAAAGGACTG-3′<br>R 5′-CCCTCAGAGTCACAAAGACCA-3′
Versican	Vcan	F 5′-GGAGGTCTACTTGGGGTGAG-3′<br>R 5′-GGGTGATGAAGTTTCTGCGAG-3′
Cspg4	NG2/Cspg4	F 5′-TCTTACCTTGGCCCTGTTGG-3′<br>R 5′-ACTCTGGTCAGAGCTGAGGG-3′
Cspg5	Cspg5	F 5′-TTGGCTTCGTCAGGCACAAT-3′<br>R 5′-GTTACACCTGCAGAAAGCCCC-3′
CD44	CD44	F 5′-CAAGTTTTGGTGGCACACAG-3′<br>R 5′-AGCGGCAGGTTACATTCAAA-3′
Phosphacan-1	Ptprz1	F 5′-CCCACTGAAGATGCCGAACT-3′<br>R 5′-GTCGTGGTAGAAACCTGGGG-3′
Ext1	Ext1	F 5’-AGCACAAGGATTCTCGCTGT-3’<br>R 5’-GGAACCAGACAGAAAGTGGC-3’
Ext2	Ext2	F 5’-ACATCCCACAGAGGCAGATT3’<br>R 5’-GATCTGTAGGGTGGCCAGAG3’
Ndst1	Ndst1	F 5′-CACACAGAACGAACTACGC-3′

		R 5′-CCCGTTGATGATCTTGTCC-3′
Ndst2	Ndst2	F 5’-TGGTCCAAGGAGAAAACCTG-3’<br>R 5’-GCAGGCTCAGGAAGAAGTGA-3’
Glce	Glce	F 5’-GCTCGCTTCAGTTTTCCTCA-3’<br>R 5’-TCTTAGTACATTTCTGGCTTCAATTC-3’
Hs2st1	Hs2st1	F 5’-TCTTGGAGAACCAGATCCAGA3’<br>R 5’-ATGGCGCTGTTCAATTTCTC3’
Hs3st1	Hs3st1	F 5’-GGAGGAGCATTACAGCCAAG-3’<br>R 5’-TTTGGGCGAAGTGAAATAGG-3’
Hs3st2	Hs3st2	F 5’-AACTACGGACGAGGACTGGA-3’<br>R 5’-ATTACCTCTGGGGCAAATCC-3’
Hs6st1	Hs6st1	F 5’-TGGCTCTTCTCTCGCTTCTC-3’<br>R 5’-GTCTAGCACACCGGGCAC-3’
Hs6st2	Hs6st2	F 5’-CCAGGCTGAGACCTTCCAG-3’<br>R 5’-TGTGGAGGATGGAGAGTTGG-3’
Sulf1	Sulf1	F 5’-CCTTGCAGGGAAGCTTCAAA-3’<br>R 5’-GCTGAGTTCTGGGAGCTTGA-3’
Sulf2	Sulf2	F 5’-GTTCCTCCCGCGATCTAGC-3’<br>R 5’-GTGTCGTGAGGATGGGATTC-3’
Hpse	Hpse	F 5’-GGCTAGAGGCTTATCTCCTGC-3’<br>R 5’-TCTTTCTTCGGAAGTCGGTT-3’
Nr3c1	Nr3c1	F 5' - ACGGTGGCAGTGTGAAATTG - 3'<br>R 5' - GACCTCCAAGGACTCTCGTT - 3'
Gapdh	Gapdh	F 5’-CGTCCCGTAGACAAAATGGT-3’<br>R 5’-TTGATGGCAACAATCTCCAC-3’
