# Supplementary material for: Multiple Administration of Dexamethasone Possesses a Deferred Long-Term Effect to Glycosylated Components of Mouse Brain
Source: Neurol Int. 2024 Jul 22;16(4):790–803. doi: 10.3390/neurolint16040058 (PMC11270268; doi:10.3390/neurolint16040058)
Supplement: Supplementary file 1 [file neurolint-16-00058-s001.zip › Supplementary Table 2 R5.pdf]

Supplementary Table 2. Expression of HS metabolism-involved genes in the mouse cerebral cortex upon multiple DXM administration. DXM doses of 1 and 2.5 mg/kg were used, the studied parameters were determined at 15, 30, 60 and 90 days after last DXM injection. Control — mouse brain tissue from untreated animals. Real-time RT-PCR analysis, intensity of the amplified DNA fragments for each gene normalized to that of GAPDH. Means ± standard deviations, ANOVA.

	**Control**	**DXM**							
		**1 mg/kg**				**2.5 mg/kg**			
		15 day	30 day	60 day	90 day	15 day	30 day	60 day	90 day
HS biosynthesis									
Ext1	3.03±0.78	3.45±0.93	1.66±0.32	1.93±1.08	3.69±1.25	3.98±1.19	3.04±2.05	2.18±0.71	3.36±1.47
Ext2	3.62±0.41	3.71±1.21	1.63±0.47	2.95±1.98	3.11±2.1	4.39±1.6	3.78±1.42	3.1±1.22	5.09±3.35
HS post-synthetic modification									
Ndst1	1.79±0.9	2.6±1.47	1.5±0.76	2.56±1.87	1.89±1.39	3.11±1.98	1.41±1.12	1.39±0.78	3.88±3.2
Ndst2	1.77±0.11	1.58±0.38	1.29±0.16	1.49±0.52	1.6±0.46	1.83±0.54	1.58±0.29	1.14±0.26	2.02±1.1
Glce	0.79±0.36	1.51±0.73	0.5±0.16	0.998±1.19	1.37±1.006	1.53±0.82	0.7±0.84	0.4±0.2	1.55±1.3
Hs2st1	1.87±0.68	2.64±0.87	1.22±0.26	1.93±1.27	2.48±1.1	2.63±1.08	2.05±1.45	1.46±0.47	3.17±1.98
Hs3st1	2.05±0.32	2.28±0.54	2.45±0.4	2.03±0.59	2.61±0.53	2.37±0.35	2.35±0.55	1.71±0.18	1.87±0.33
Hs3st2	0.01±0.003	0.01±0.002	0.03±0.01	0.11±0.13	0.02±0.009	0.01±0.002	0.08±0.12	0.04±0.03	0.19±0.008
Hs6St1	4.73±0.49	4.33±1.12	5.17±1.77	6.85±2.4	4.83±1.53	5.24±1.53	5.74±2.18	4.3±0.99	6.22±3.2
Hs6St2	0.21±0.98	0.08±0.03	1.54±1.09	1.48±1.7	0.46±0.47	0.18±0.08	6.36±9.89	2.22±1.41	0.58±0.25
HS desulfation and degradation									
Sulf1	0.16±0.07	0.33±0.19	0.16±0.05	0.27±0.24	0.36±0.27	0.26±0.15	0.18±0.2	0.11±0.059	0.49±0.4
Sulf2	0.48±0.17	0.72±0.35	0.73±0.4	0.82±0.49	0.59±0.4	0.78±0.39	0.43±0.38	0.57±0.06	1.23±0.81
Hpse	0.13±0.1	0.08±0.03	0.09±0.05	0.08±0.06	0.12±0.04	0.11±0.075	0.28±0.28	0.04±0.013	0.11±0.025
