# Supplementary material for: Multiple Administration of Dexamethasone Possesses a Deferred Long-Term Effect to Glycosylated Components of Mouse Brain
Source: Neurol Int. 2024 Jul 22;16(4):790–803. doi: 10.3390/neurolint16040058 (PMC11270268; doi:10.3390/neurolint16040058)
Supplement: Supplementary file 1 [file neurolint-16-00058-s001.zip › Supplementary Table 3 R5.pdf]

Supplementary Table 3. Expression of PG core proteins-coding genes in the mouse cerebral cortex upon multiple DXM administration. DXM doses of 1 and 2.5 mg/kg were used, the studied parameters were determined at 15, 30, 60 and 90 days after last DXM injection. Control — mouse brain tissue from untreated animals. Real-time RT-PCR analysis, intensity of the amplified DNA fragments for each gene normalized to that of GAPDH. Means ± standard deviations, ANOVA.

	**Control**	**DXM**							
		**1 mg/kg**				**2.5 mg/kg**			
		15 day	30 day	60 day	90 day	15 day	30 day	60 day	90 day
HSPG									
Sdc-1	0.25± 0.12	0.12±0.02	0.27±0.14	0.31±0.09	0.27±0.17	0.2±0.04	0.61±0.62	0.23±0.1	0.32±0.16
Sdc-3	2.61±1.21	3.35±1.58	1.78±0.8	3.41±2.21	1.74±1.13	3.77±2.13	2.44±1.93	2.92±1.29	6.96±5.64
Gpn-1	0.99±0.24	0.87±0.36	0.67±0.25	1.19±0.52	0.61±0.3	1.08±0.48	0.97±0.53	0.83±0.42	1.24±0.85
Hspg2	0.03±0.02	0.03±0.02	0.04±0.03	0.04±0.04	0.02±0.01	0.04±0.03	0.02±0.01	0.02±0.01	0.11±0.08
CSPG									
Dcn	12.19±8.51	18.76±6.21	14.69±3.83	20.46±12.59	19.05±3.36	12.31±5.66	18.31±6.12	15.37±0.16	21.64±6.08
Bgn	0.45±0.14	0.47±0.13	0.84±0.4	1.29±1.00	0.73±0.43	0.61±0.22	0.51±0.25	0.33±0.11	0.85±0.64
Lum	0.02±0.01	0.01±0.01	0.03±0.02	0.06±0.06	0.04±0.03	0.04±0.02	0.06±0.05	0.04±0.03	0.05±0.05
Bcan	7.16±0.79	6.24±1.81	6.1±0.77	11.68±7.75	10.36±5.55	10.89±4.63	16.43±3.74	12.42±7.19	13.06±10.97
Ncan	2.31±1.54	2.94±1.96	0.8±0.38	2.02±2.04	1.2±0.73	4.02±2.81	1.71±1.12	1.64±0.06	5.71±5.09
Acan	0.07±0.02	0.05±0.01	0.56±0.42	0.34±0.27	0.05±0.03	0.07±0.04	0.2±0.26	0.1±0.04	0.11±0.03
Vcan	0.12±0.07	0.18±0.1	0.44±0.3	0.4±0.29	0.12±0.08	0.16±0.06	0.33±0.21	0.2±0.09	0.53±0.31
Cspg/NG2	0.7±0.07	0.5±0.15	0.35±0.05	0.65±0.49	0.48±0.28	0.67±0.23	0.68±0.19	0.59±0.41	0.87±0.6
Cspg5	99.24±17.26	84.0±10.92	113.95±27.8	87.86±10.31	97.35±17.46	98.91±12.08	121.95±35.17	79.57±21.20	107±17.91
CD44	0.28±0.12	0.76±0.42	0.12±0.03	0.37±0.4	0.84±0.49	0.44±0.2	0.38±0.42	0.18±0.1	0.98±0.9
Ptprz1	6.49±5.04	14.01±9.06	0.84±0.1	10.32±18.69	11.72±13.01	12.66±9.18	6.97±12.0	1.97±1.26	29.44±27.40
